# Bangladeshi medicinal plant dataset

**DOI:** 10.1016/j.dib.2023.109211

**Published:** 2023-05-07

**Authors:** Bijly Borkatulla, Jannatul Ferdous, Abdul Hasib Uddin, Prince Mahmud

**Affiliations:** a Department of Computer Science & Engineering, Khwaja Yunus Ali University, Enayetpur, Chouhali, Sirajganj 6751, Bangladesh; b Department of Computer Science & Engineering, Jannat Ara Henry Science & Technology College, Sirajganj, Bangladesh

**Keywords:** Medicinal plant, Image classification, Image processing, DenseNet201, Feature visualization

## Abstract

Medicinal plants have been used to treat diseases since ancient times. Plants used as raw materials for herbal medicine are known as medicinal plants [2]. The U. S. Forest Service estimates that 40% of pharmaceutical drugs in the Western world are derived from plants [1]. Seven thousand medical compounds are derived from plants in the modern pharmacopeia. Herbal medicine combines traditional empirical knowledge with modern science [2]. A medicinal plant is considered an important source of prevention against various diseases [2]. The essential medicine component is extracted from different parts of the plants [8]. In underdeveloped countries, people use medicinal plants as a substitute for medicine. There are various species of plants in the world. Herbs are one of them, which are of different shapes, colors, and leaves [5]. It is difficult for ordinary people to recognize these species of herbs. People use more than 50000 plants in the world for medicinal purposes. There are 8000 medicinal plants in India with evidence of medicinal properties [7]. Automatic classification of these plant species is important because it requires intensive domain knowledge to manually classify the proper species. Machine learning techniques are extensively used in classifying medicinal plant species from photographs, which is challenging but intriguing to academics. Artificial Neural Network classifiers’ effective performance depends on the quality of the image dataset [4]. This article represents a medicinal plant dataset: an image dataset of ten different Bangladeshi plant species. Images of medicinal plant leaves were from various gardens, including the Pharmacy Garden at Khwaja Yunus Ali University and the Khwaja Yunus Ali Medical College & Hospital in Sirajganj, Bangladesh. Images were collected by taking pictures with high-resolution mobile phone cameras. Ten medicinal species, 500 images per species are included in the data set, namely, Nayantara (*Catharanthus roseus*), Pathor kuchi (*Kalanchoe pinnata*), Gynura procumbens (*Longevity spinach*), Bohera (*Terminalia bellirica*), Haritaki (*Terminalia chebula*), Thankuni (*Centella asiatica*), Neem (*Azadirachta indica*), Tulsi (*Ocimum tenniflorum*), Lemon grass (*Cymbopogon citratus*), and Devil backbone (*Euphorbia tithymaloides*). This dataset will benefit researchers applying machine learning and computer vision algorithms in several ways. For example, training and evaluation of machine learning models with this well-curated high-quality dataset, development of new computer vision algorithms, automatic medicinal plant identification in the field of botany and pharmacology for drug discovery and conservation, and data augmentation. Overall, this medicinal plant image dataset can provide researchers in the field of machine learning and computer vision with a valuable resource to develop and evaluate algorithms for plant phenotyping, disease detection, plant identification, drug development, and other tasks related to medicinal plants.

**Specifications Table**

**Table:** 

Subject	Computer Science, Botany

Specific subject area	Computer vision, Image classification, Image processing, Machine learning.
Type of data	Plant and leaf Images.
How the data were acquired	Images were captured using a 13 megapixel (409ppi) smartphone camera on the Redmi Note 8 features (resolution: 1080 × 2340 pixels). Other smartphones with 8-megapixel (269ppi) cameras include the Xiaomi 7 (resolution: 720 × 1520 pixels) and the Samsung Galaxy A51 32 megapixel camera (resolution: 1080 × 2400 pixel).
Data format	PNG, Raw.
Description of data collection	Collected the images directly using several smartphones with different configurations as described in the “How the data were acquired” row. This dataset has 5000 images from ten classes.
Data source location	Pharmacy Garden, Khwaja Yunus Ali University and Khwaja Yunus Ali Medical College & Hospital, Sirajganj, Bangladesh.
Data accessibility	Repository name: Kaggle Data identification number [[10], [11]]: 10.34740/KAGGLE/DSV/4510170 Direct URL to data: 10.34740/KAGGLE/DSV/4510170

## Value of the Data


 

• This dataset includes the identification of the following ten Bangladeshi medicinal plant species, namely, *Catharanthus roseus, Kalanchoe* pinnata, *Longevity spinach,Terminalia bellirica,Terminalia chebula, Centella asiatica, Azadirachta indica, Ocimum tenniflorum, Cymbopogon citratus*, and *Euphorbia tithymaloides*.
• The information gathered is of the high quality and valuable, with the goal of serving as content for data analysis.
• The dataset may prove helpful in testing image recognition classifiers for the identification of various medicinal plants.
  Using the dataset's images of medicinal plant leaves, classification algorithms can be trained, tested, and validated.
• The data can be used for a variety of machine-learning applications, including image classification and image detection.

## 1 Objective

The objective behind building this dataset was to reduce manual work and increase efficiency by the automatic identification of medicinal plants using image processing techniques. Additionally, the images for this dataset were collected in a controlled environment (by placing the leaves on white papers and providing adequate lighting) to ensure high-quality pictures for future use. Moreover, a challenging multiclass image dataset will allow the researchers to test several features and evaluate how well they perform. Also, another aim of this dataset is to identify and classify medicinal plants without any human assistance. It will help develop Machine and Deep Learning classifiers for effective medicinal plant classification based on the distinctive characteristics of these plants. This dataset can be used to validate the data as well as perform the necessary calibration of the data. These datasets can also be used for comparing the accuracy of different models. These data can also be used to develop a new system.

## 2 Data Description

Identifying proper medicinal plant species for the corresponding disease is one of the many uses for medicinal plant classification. Manual identification of plants is a time-consuming process and requires expert help [9]. It is necessary for the greater good of humanity to solve this problem to identify and classify medicinal plants automatically. Hence, automatic classification and identification of medicinal plants are valuable in image processing research. Feature extraction is a significant step in the identification of medicinal plants. For this purpose, high-quality images are required.

The most famous architecture for classifying images using visual data is CNN. Deep learning methods use convolutional layers for automatic feature extraction [8]. Medicinal plant identification and classification are performed using image processing, machine learning, and computer vision techniques [3].

Medicinal plants: This data set has five thousand images from ten different classes of medicinal plant species, i.e., Nayantara (*Catharanthus roseus*), Pathor kuchi (*Kalanchoe pinnata*), Gynura procumbens (*Longevity spinach*), Bohera (*Terminalia bellirica*), Haritaki (*Terminalia chebula*), Thankuni (*Centella asiatica*), Neem (*Azadirachta indica*), Tulsi (*Ocimum tenniflorum*), Lemon grass (*Cymbopogon citratus*), and Devil backbone (*Euphorbia tithymaloides*). The species names were authenticated by a botanist from the Department of Botany of a govt. College. As for several of these species hold compound phyllotaxy, the objects were significantly big. Hence, to capture the entire object, we considered large images for our dataset. Images were captured using a 13-megapixel (409ppi) smartphone camera on Redmi Note 8. Other smartphones Xiaomi 7 megapixel-8 (269ppi) cameras and the Samsung Galaxy A51 with 32-megapixel (405ppi) camera. The images were collected from the Pharmacy Garden, Khwaja Yunus Ali University, and the Khwaja Yunus Ali Medical College & Hospital in Sirajganj, Bangladesh provided the images for the dataset. Ten species of medicinal plant leaf images were used, five hundred images per species. Five thousand images total, from which 3500 are training images, 1000 are testing images, and 500 are validation sets. The dataset contains only images.

**Fig. 1:**
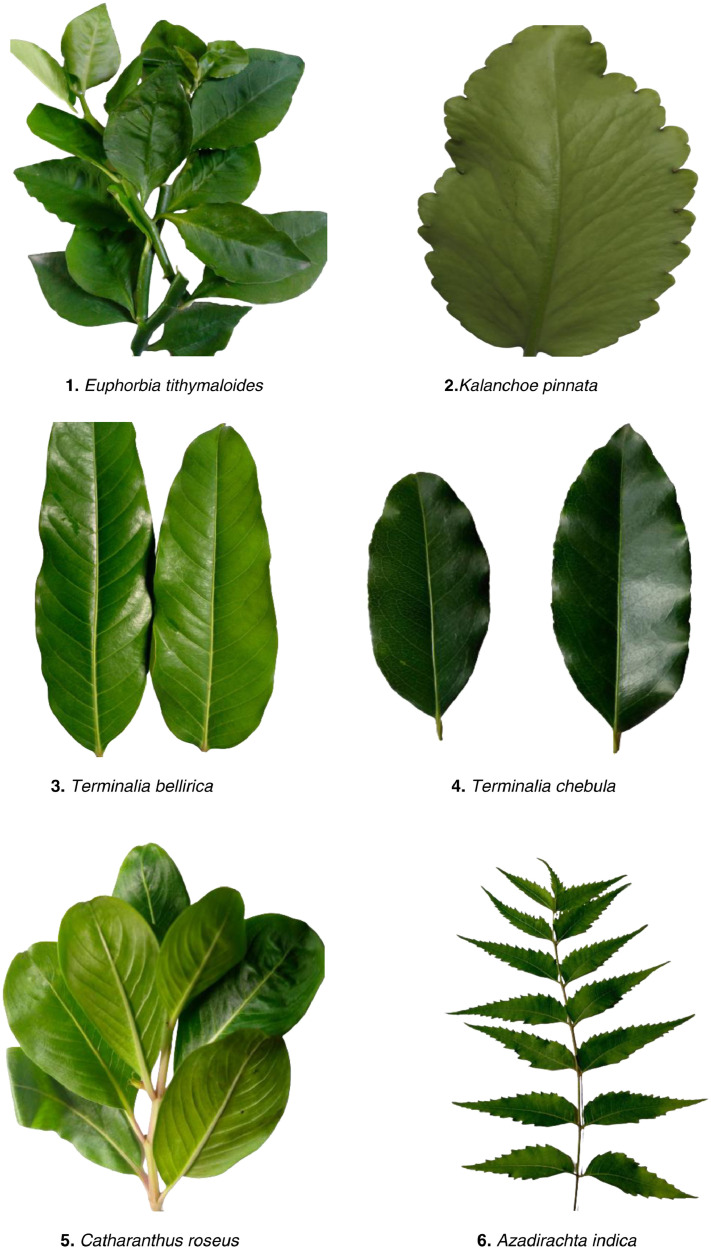

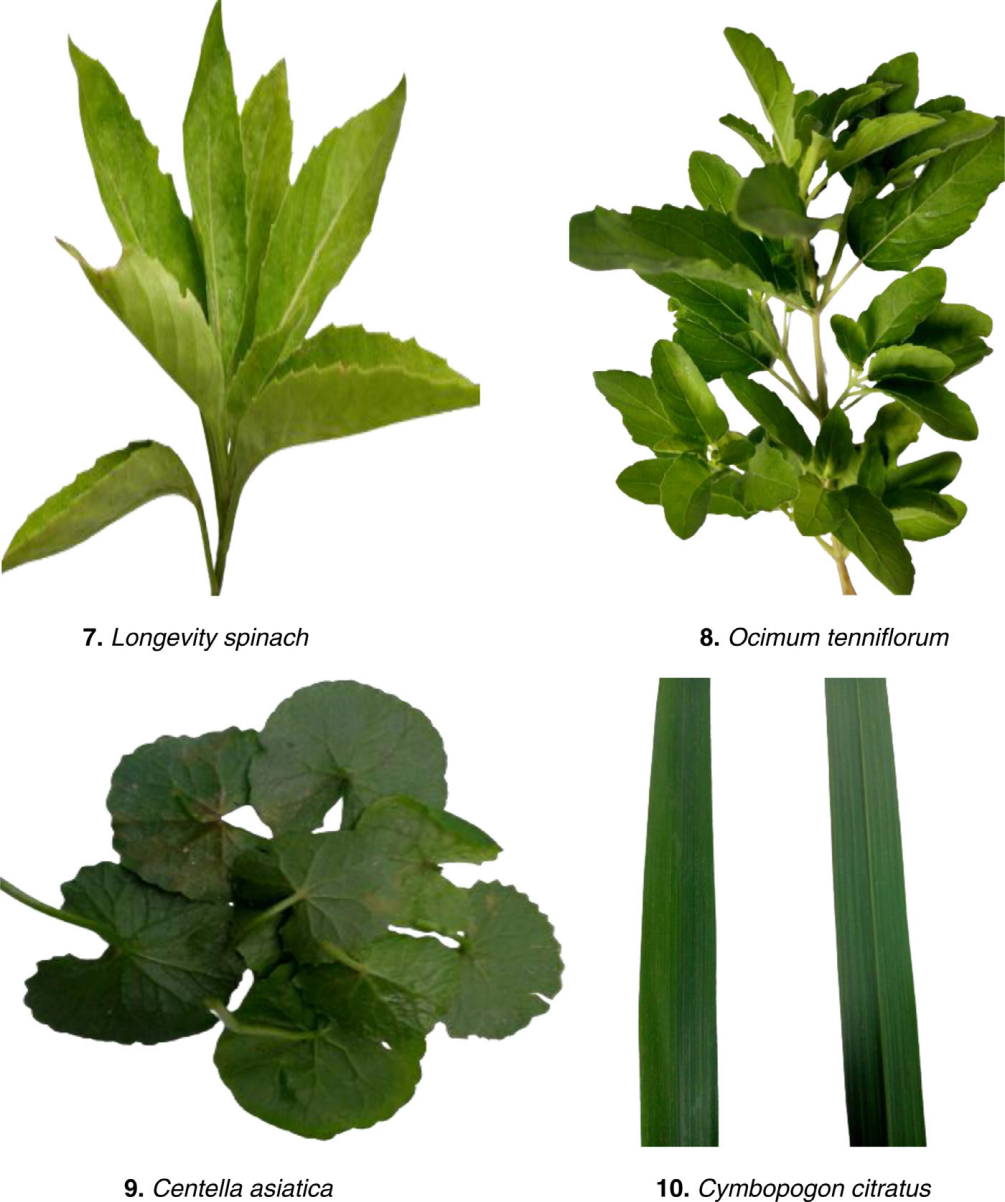
Example leaves images of 10 different medicinal plants.

**Fig. 2:**
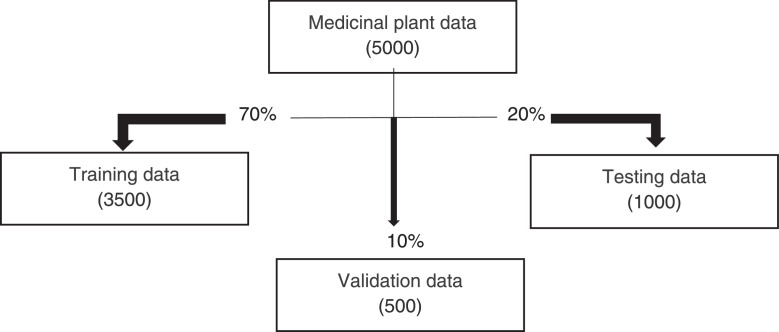
Data distribution.

**Fig. 3:**
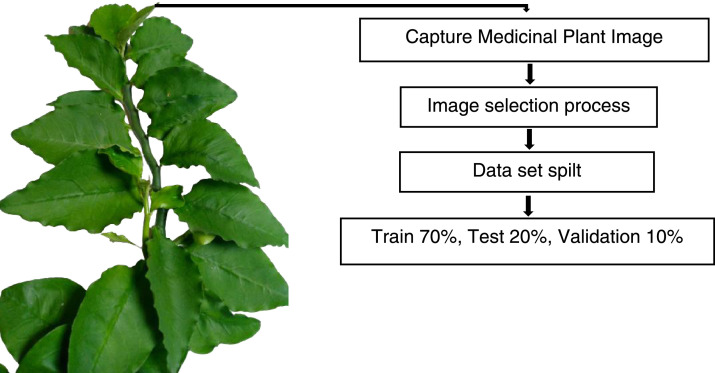
Image processing steps

**Fig. 4:**
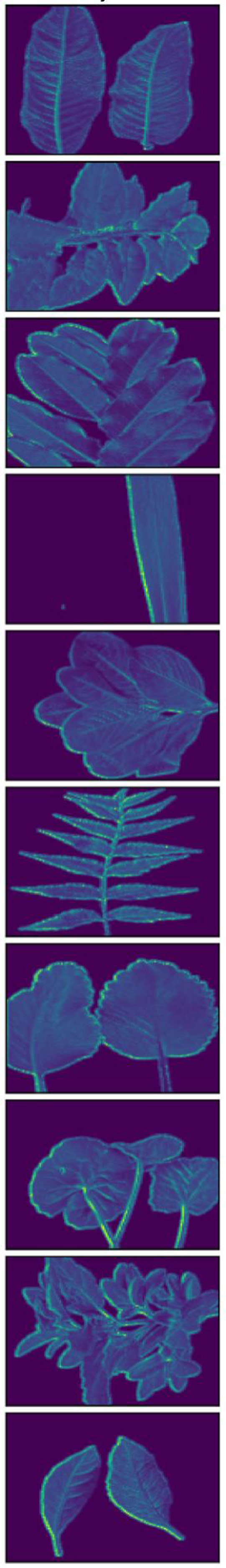
Feature visualization

The images of ten medicinal plant leaves are shown in Fig. 1. The processing time for images is presented in Table 1. The detailed characteristics of the medicinal plant dataset with regarding several plant properties are included specific details regarding various plant leaves and other related information is shown in Table 2. The image distribution data set has been described in Fig. 2. The image processing steps are illustrated in Fig. 3. Fig. 4 visualizes the features extracted by the fourth layer (first convolutional layer) of the final trained DenseNet201 model.

**Table 1:** The steps of data collection have been segmented into tabular forms.

Process	Time	Work
Image capture	July to August 2022	The images were acquired directly the pictures were taken using a mobile phone. Approximately 6,000 images were captured of which 5,000 clear images were selected in the data set.
Processing of dataset	September to October	Images are arranged in different folders such as train, test, and validation. Model implementation done (ResNet50, DenseNet201, VGG16, and InseptionV3).

**Table 2:** Morphological features of various plant species are determined automatically from the Medicinal Plant dataset.

Species	Characters		
	Color	Body Shape	Leaves shape
*Catharanthus roseus*	Glossy green	Plant 1 m (39in) tall.	Oval or oblong, smooth, opposite leaves [5].
*Kalanchoe pinnata,*	Green	Simple stem base; the upper 10–30 cm (4–12 in) of the leaves are imparipinnate.	The leaves are oval in form, thick, meaty, and frequently reddish.
*Longevity spinach*	Green	simple stem bases 15-20cm (4-8 in) in length	Diamond-shaped rhombic designs.
*Terminalia bellirica*	Green and deep grey in color	A big deciduous tree, Bohera.	Broad obovate-elliptic to obovate-oblong, and occasionally narrowly oblanceolate leaves.
*Terminalia chebula*	Green & Deep grey in color	The Haritaki is a big deciduous tree that grows to a height of 30 to 40 meters.	The leaves are generally obovate-elliptic to obovate-oblong.
*Centella asiatica*	Color ranges from reddish-green to green.	The stems are thin, spreading stolons.	Reniform round-shape[6]
*Azadirachta indica*	Bright green	Neem is a sizable deciduous tree with a height of 15 to 30 meters (40 to 100 feet), a round, beautiful crown, and thick, deeply furrowed bark.	They are elongated to oblong and medium to large in size.
*Ocimum tenniflorum*	Green	The stems have simple teeth and are hairy. 30–60 cm tall with stems that are hairy [6].	Leaves have an oval shape [5].
*Cymbopogon citratus*	plain, bluish-green	Typically, the blades are 18–36 inches long.	linear Long, thin, and slender
*Euphorbia tithymaloides*	Green/Gray	2-foot tall, robust stems with stalks.	Lance Shape Leaves

### 2.1 Experimental Design, Materials, and Methods

All the pre-processing techniques used on the data to produce the final dataset are discussed in this area of the experimental design, materials, and methods.

## 3 Experimental Design Materials: Tools and Devices

Leaf sampling take easy and taking photos is convenient. Therefore, images were captured using a 13-megapixel (409ppi) Redmi Note 8 smartphone camera, as well as other smartphones such as Xiaomi 7 megapixel-8 (269ppi) cameras and the Samsung Galaxy A51 with 32-megapixel (405ppi) camera. The images were collected from the Pharmacy Garden, Khwaja Yunus Ali University, and the Khwaja Yunus Ali Medical College & Hospital in Sirajganj, Bangladesh.

## 4 Experimental Design

The Khwaja Yunus Ali University, Khwaja Yunus Ali Medical College Pharmacy Garden, and local plant gardens in and near Enayetpur, Sirajganj provided the images for the dataset. Medicinal plant images were taken from July to August using a mobile phone. A total of five thousand images have been collected. The dataset was then processed from September to October. Five thousand images Table 1. Ten medicinal plants name and scientific names are discussed about plant color plant body shape leaf shape and each leaf shape are described separately in Table 2. The image distribution data set has been described in Fig. 2 First five thousand images are divided into three parts train, test, and validation. The 5000 images are split into 3500 for training, 1000 for testing, and 500 for validation. The image processing step has been described in Fig. 3 first the image of the medicinal plant is taken directly, then noisy images were discarded and the clearest ones were selected, then the data set is spilt and the train, test and validation are divided into three parts.

## 5 Methods

We have applied the ImageNet pre-trained ResNet50, DenseNet201, VGG16, and InceptionV3 models to our dataset. The batch size was 32. Train and validation data were shuffled on each epoch while training. The corresponding learning rate was 0.0001, learning rate decay was 0.00001. We utilized categorical cross entropy as the loss function for learning and SoftMax classifier for the classification purpose. RMSprop was used as the optimizer. We have trained each model until encountering no improvements on validation loss for consecutive 10 epochs. The performances of these models in term of accuracy are 72%, 97%, 96%, and 95%, respectively.

As DenseNet201 performs the best, we considered this model for feature visualization. Fig. 4 visualizes the features extracted by the fourth layer (first convolutional layer) of the final trained DenseNet201 model. The first convolutional layer was chosen for feature visualization as it produces easily interpretable results for human understanding. As layers go deeper, number of filters increases and images dimension decreases. Hence, visualizations of deeper layers become difficult to understand. From Fig. 4, we can clearly see that the geometric shapes of the leaves are considered as the primary feature by the neural network. Also, first degree of venation is extracted as another important feature, where possible.

## Ethics Statements

Neither human participants nor animal experiments are used in the work given here. It only contains photographs that were obtained by the author and does not include those that were gathered from social media sites. For performing this work, we were not given any funding.

## CRediT authorship contribution statement

**Bijly Borkatulla:** Data curation, Writing – original draft. **Jannatul Ferdous:** Data curation. **Abdul Hasib Uddin:** Conceptualization, Supervision, Methodology, Writing – review & editing. **Prince Mahmud:** Supervision, Writing – review & editing.

## Declaration of Competing Interest

The authors declare that they have no known competing financial interest or personal relationships that could have appeared to influence the work reported in this paper.

## Data Availability

Medicinal Plant Raw (Original data) (Kaggle).
